# Suicide, quality of life, and psychotropic medication use in women with breast cancer

**DOI:** 10.1590/0034-7167-2024-0573

**Published:** 2026-08-03

**Authors:** Leonardo Felipe Ferreira de Carvalho, Adriana Inocenti Miasso

**Affiliations:** I Universidade de São Paulo. Ribeirão Preto, São Paulo, Brazil

**Keywords:** Cross-Sectional Studies, Suicide, Psychotropic Drugs, Quality of Life, Oncology., Estudios Transversales, Suicidio, Psicotrópicos, Calidad de Vida, Oncología.

## Abstract

**Objectives:**

to identify factors associated with suicide risk, quality of life, and psychotropic medication use among women with breast cancer treated at an oncology hospital.

**Methods:**

a cross-sectional, descriptive, correlational study with a quantitative approach was conducted in the mastology outpatient clinic of a specialized oncology hospital. A total of 102 women participated and completed specific questionnaires. Linear and logistic regression models were used to assess the association between psychotropic use, suicide risk, and quality of life.

**Results:**

15.7% of participants presented suicide risk, and 23.6% used psychotropic medications, mainly benzodiazepines and antidepressants. Non-Catholic women and those in stage III showed higher psychotropic consumption. Quality of life was lower in physical and psychological domains, and psychotropic use was associated with poorer scores across all domains.

**Conclusions:**

the findings highlight the importance of early identification of suicide risk and the development of personalized emotional support strategies for women with breast cancer.

## RESUMEN

**Objetivos:**

identificar factores asociados al riesgo suicida, calidad de vida y consumo de psicotrópicos en mujeres con cáncer de mama atendidas en hospital oncológico.

**Métodos:**

estudio transversal, descriptivo y correlacional, con enfoque cuantitativo, realizado en el consultorio de mastología de hospital especializado en oncología. Participaron 102 mujeres que respondieron a cuestionarios específicos. Se utilizaron modelos de regresión lineal y logística para evaluar la asociación entre el uso de psicotrópicos, riesgo suicida y calidad de vida.

**Resultados:**

15,7% de las participantes presentaron riesgo suicida y el 23,6% utilizaban psicotrópicos, principalmente benzodiacepinas y antidepresivos. Mujeres no católicas y aquellas en estadio III de la enfermedad mostraron un mayor consumo de psicotrópicos. Calidad de vida fue menor en los dominios físico y psicológico, y el uso de psicotrópicos se asoció con peores puntajes en todos los dominios.

**Conclusiones:**

los hallazgos destacan la importancia de la identificación temprana del riesgo suicida y estrategias personalizadas de apoyo emocional para mujeres con cáncer de mama.

## INTRODUCTION

Suicide is one of the leading causes of death globally, representing a significant public health concern. According to recent estimates from the World Health Organization (WHO), more than 700,000 people die by suicide each year, which equates to one death every 40 seconds, highlighting the urgent need for effective prevention strategies, especially in vulnerable populations such as women diagnosed with breast cancer^(1)^.

Breast cancer is the most common type of cancer among women, with an increasing incidence driven by population aging and lifestyle changes^(2)^.

The Brazilian National Cancer Institute projects that Brazil will register 73,610 new cases of the disease in 2024, with a rate of 66.54 cases per 100,000 women^(3,4)^. Research shows that a breast cancer diagnosis causes not only significant physical suffering, but also severe psychological distress, especially in the first few years after diagnosis, when patients face intense emotional and psychological conflict, which has been suggested to increase the risk of suicide^(5)^.

The psychological burden faced by women with breast cancer is multifaceted. Anxiety about impending mortality, changes in body image due to surgeries and treatments, and stigma associated with the disease are factors that significantly contribute to an increased risk of suicide^(6-11)^. Furthermore, depression and anxiety are prevalent in this population, with significantly higher rates than in the general population. Depression, in particular, is a known risk factor for suicide and can negatively affect treatment adherence and quality of life^(7,11)^.

Breast cancer management often involves psychotropic medication use to treat secondary psychological symptoms such as depression and anxiety^(12,13)^. These medications, while effective in improving quality of life, present challenges related to drug interactions and potential side effects. Furthermore, stigma associated with psychotropic medication use can negatively influence patients’ willingness to seek treatment^(14)^.

The projected increase in breast cancer cases could exceed 2 million annually worldwide by 2025. This highlights the need for integrative health approaches that not only treat the physical disease but also offer comprehensive emotional and psychological support^(15,16)^.

Intervention strategies that combine pharmacological treatment with psychosocial therapies have shown promising results in reducing symptoms of depression and anxiety, improving patients’ quality of life^(17,18)^.

## OBJECTIVES

To identify factors associated with suicide risk, quality of life, and psychotropic medication use among women with breast cancer treated at an oncology hospital.

## METHODS

### Ethical aspects

The study respected the ethical precepts of research with human beings, having been approved by the Research Ethics Committee of the proposing institution. The subjects were informed about anonymity and the freedom to accept or refuse participation in the research. All participants who agreed to participate in the study signed the Informed Consent Form.

### Theoretical framework

The relationships between specific variables were analyzed according to the frameworks described in WHO guidelines for research in mental health and quality of life, and standardized instruments such as the Mini International Neuropsychiatric Interview (MINI) and the World Health Organization Quality of Life - brief version (WHOQOL-bref).

### Study design

This is an observational, cross-sectional study with a quantitative approach and exploratory character, conducted in accordance with Strengthening the Reporting of Observational Studies in Epidemiology guidelines. The cross-sectional design allows for the simultaneous analysis of exposure and outcome at a given time point, enabling the identification of associations among variables, although it does not allow for inferring causality.

### Study setting

The study was conducted in the mastology outpatient clinic of an oncology hospital located in southeastern Brazil. This clinic provides patient care from diagnosis, through surgical, chemotherapeutic, radiotherapeutic, and rehabilitation treatments, and, if necessary, palliative and end-of-life care. The service has an interdisciplinary team that includes radiologists, mastologists, nuclear medicine physicians, geneticists, oncologists, radiation oncologists, pathologists, nurses, physiotherapists, nutritionists, psychologists, and all other professionals who can contribute to the ideal treatment for each patient.

### Population and sample

The study population consisted of women who were being monitored at the mastology outpatient clinic of a public oncology hospital in southeastern Brazil. To determine the sample size, a sample size calculation was performed by a statistician from the Epidemiology and Biostatistics Center of the aforementioned hospital. Sample size calculation was performed using as a reference the data from a study^(19)^ that applied the WHOQOL-bref to women with breast cancer. A confidence level of 95% and a tolerable absolute error of 1 point were considered in the scores of the aforementioned instrument, thus estimating the need for a minimum sample of 102 participants, a number that was reached during the data collection period. This calculation followed the formula n=(Z·σ/d)^2, com Z=1.96, the absolute error d=1 point and the standard deviation of the most variable domain of the WHOQOL-bref in the reference study justify the minimum sample size of 102. This procedure ensured greater precision in estimating the mean. Even so, it is recognized that this is an exploratory study, subject to the limitations inherent in the number of participants.

Inclusion criteria comprised being a woman diagnosed with any type of breast cancer, being 18 years of age or older, being able to communicate verbally in Portuguese, and being followed up at the mastology outpatient clinic of the hospital under study. Exclusion criterion comrpsied not answering the questions in their entirety in the research instruments.

### Methodological procedures

For data collection, visits were made to the mastology outpatient clinic between March and July 2024. Patients who attended the clinic for care and who met the inclusion criteria were invited to participate in the research. Participants who agreed to participate received detailed information about the study, and after signing the Informed Consent Form, participated in an interview conducted by a researcher from the study. The interviews were conducted in the mastology outpatient clinic’s consulting rooms, with an average duration of 30 minutes. A structured questionnaire, developed by the researchers and with content validation by judges, and previously validated instruments were used to conduct the interviews.

The questionnaire used addressed sociodemographic, clinical, and psychotropic medication use variables, including age, education level, marital status, religion, employment/occupation status, individual and/or family monthly income, number of residents in the household, primary cancer site, duration of cancer treatment, history of cancer surgeries, presence of other illnesses, and continuous non-psychotropic medication use. For data collection regarding the “psychotropic medication use”, outcome variable the question “Do you use psychotropic medications?” was included in this instrument, with possible answers of “yes” and “no”.

To assess suicide risk, the MINI version 5.0.0, Module C - Suicide Risk, was used. The MINI is a structured and internationally validated instrument, adapted to the Brazilian population^(20)^. It exhibits high sensitivity and specificity, as well as good reliability, making it suitable for use in clinical settings^(20)^, including cancer patients, due to its ability to quickly and standardizedly identify relevant emotional symptoms. The questionnaire is organized into independent diagnostic modules, designed to optimize the instrument’s sensitivity. Responses consist of a dichotomous variable: yes or no. The modules are identified by letters, and each letter corresponds to a diagnostic category^(20)^. Considering the objectives of this research, Module C - Suicide Risk - was used.

Quality of life was assessed using the WHOQOL-bref, which encompasses four domains: physical; psychological; social relationships; and environment. The WHOQOL-bref was developed by the WHO in partnership with 20 research centers located in 18 countries, and was validated in Brazil^(21)^, showing high consistency and good construct and discriminant validity. The results obtained in the instrument are classified according to the average of responses, according to the following scale: needs improvement (when it is 1 to 2.9); fair (3 to 3.9); good (4 to 4.9); and very good (5). An integrative review investigating the WHOQOL-BREF’s reliability in research with women diagnosed with breast cancer indicated that the questionnaire presents adequate levels of internal consistency. The results showed that Cronbach’s alpha values for the different quality of life domains remain stable, regardless of the stage of the disease, reinforcing the usefulness of the instrument in this specific population^(21)^.

Participant confidentiality was ensured by conducting the interviews in a private location, coding the data without identifying names, and storing the information in a secure electronic environment with access restricted to the research team.

Data collection was interrupted when the planned sample size of 102 participants was reached. Patients identified as being at risk of suicide were referred to the Psycho-Oncology Department of the hospital under study for medical assessment and psychological and psychiatric follow-up.

### Data analysis

For data analysis, a quantitative approach was used. Data were collected using a computer during the interview and stored using the REDCAp platform. Subsequently, the data were transferred to the Statistical Package for the Social Sciences version 21.5 for analysis. The researchers committed to collecting and storing the data in accordance with the General Data Protection Law. To address suicide risk and psychotropic medication use as dependent variables, univariate analyses (chi-square test and, in cases of categories with fewer than five individuals, Fisher’s exact test was used) and multivariate logistic regression were performed. For quality of life analysis, Student’s t-test and multiple linear regression were used. Associations with p≤0.05 were considered significant. In addition to comparisons between groups, we explored the correlation between suicide risk and the WHOQOL-bref domains using point-biserial correlation; when indicated, we used Spearman’s correlation.

## RESULTS

One hundred and two women with breast cancer participated in the study. The majority were in the 41-59 age range (53.9%), had completed high school (51%), had a partner (67.6%), were unemployed (62.7%), had an income equal to or less than three minimum wages (62.7%), and did not live alone (88.2%). There was a higher percentage of participants from the Northeast region (35.3%) and non-Catholic (50%). Concerning clinical characteristics, it was found that the highest percentage of participants were in stage I of the disease (44.6%), with comorbidities (50.9%), and using non-psychotropic medications (55.9%) (Table 1).

**Table 1:** Sociodemographic and clinical characteristics of women with breast cancer treated at a mastology outpatient clinic of a Brazilian oncology hospital (N=102)

Variáveis	n	%
Age (years)		
18-40	3	2.9
41-59	55	53.9
Equal to or greater than 60	43	42.2
Missing	1	0.9
Total	102	100
Region		
North	22	21.6
Northeast	36	35.3
Midwest	4	3.9
Southeast	32	31.4
South	5	4.9
Missing	3	2.9
Total	102	100.0
**Education**		
Illiterate	2	2.0
Elementary school	21	20.6
Middle school	52	51.0
Higher education	27	26.5
Total	102	100
**Marital status**		
No partner	33	32.4
With partner Missing	69	67.6
Total	102	100
**Religion**		
Catholic	49	48.0
Non-Catholic	51	50.0
Missing	2	2.0
Total	102	100.0
**Occupation**		
Unoccupied	64	62.7
Occupied	36	35.3
Missing	2	2.0
Total	102	100.0
**Income**		
≤ 3 minimum wages	64	62.7
> 3 minimum wages	38	37.3
Total	102	100.0
**Lives alone**		
No	90	88.2
Yes	9	8.8
Missing	3	3.0
Total	102	100.0
**Stage**		
I	45	44.6
II	40	39.6
III	17	15.8
Total	102	100.0
**Comorbidities**		
No	45	44.1
Yes	52	50.9
Missing	5	5.0
Total	102	100.0
**Non-psychotropic medication use**		
No	45	44.1
Yes	57	55.9
Total	102	100.0

This study, using the MINI, found that 15.7% of participants were classified as being at risk for suicide, while 43.8% were not at risk. For the chi-square test, the “suicide risk” variable was classified as “at risk” and “not at risk”. Univariate analysis showed that none of the sociodemographic variables investigated were associated with suicide risk. In relation to clinical and therapeutic characteristics, there was a significant association between psychotropic medication use and suicide risk (p=0.001). It was found that, among women at risk of suicide, the majority were using psychotropic medications (56.2%).

Table 2 presents a logistic regression model investigating the relationship between suicide risk in breast cancer patients and psychotropic medication use. The results show a significant correlation between psychotropic medication use and a higher risk of suicide. There was an Odds Ratio (OR) of 7.92 and a 95% Confidence Interval (CI) between 2.5 and 25.3 (p=0.0005). This indicates that patients who use psychotropic medications have a higher probability of suicide than those who do not.

**Table 2:** Logistic regression model for analyzing factors associated with suicide risk in breast cancer patients

	Variable	Category	OR	95%CI	*p* value
**Suicide risk**	Psychotropic medication use	No	Ref	Ref	Ref
		Yes	7.92	2.5 - 25.3	0.0005

*OR - Odds Ratio; 95%CI - 95% Confidence Interval.*

It was found that the mean quality of life scores were lower in the physical (3.7) and psychological (3.8) domains, and higher in the social relations (4.0) and environment (4.0) domains. In the physical and psychological domains, most women were classified as having a fair quality of life (72.5% and 69.6%, respectively). In the social relations and environment domains, most were classified as having a good quality of life (72.5% in both domains) (Table 3).

**Table 3:** Analysis of performance categorized according to the World Health Organization Quality of Life - brief version domains and by average

Variables	Needs improvement		Fair		Good		Very good		Mean
	n	%	n	%	n	%	n	%	
Quality of life - performance									
Physical	7	6,9	74	72.5	21	20.6	0	0	3.7
Psychological	6	5,9	71	69.6	22	21.6	3	2.9	3.8
Social relationships	2	2,0	15	14.7	74	72.5	11	10.8	4.0
Environment	3	2,9	22	21.6	74	72.5	3	2.9	4.0

In the analysis of the association between suicide risk and quality of life domains, no statistically significant differences were observed. However, some descriptive patterns were identified, suggesting that patients with a higher suicide risk tended to report worse scores in certain domains. A linear regression model was performed to identify the impact of independent variables on the different WHOQOL-bref domains. In the model, all variables that showed a significant association (p<0.05) in the univariate analysis were included. In the physical domain, there was an association with the “stage of breast cancer (p=0.008)” and “psychotropic medication use” variables (p=0.001). In the psychological domain, an association was found with education level (p=0.034), stage of breast cancer (p=0.002), comorbidities (p=0.020), and psychotropic medication use (p<0.001). In the social relations domain, the “stage of breast cancer” (p=0.031) and “psychotropic medication use” (p=0.022) variables were associated. There was a significant association between the quality of life classification in the environmental domain and the “psychotropic medication use” (p=0.006) variable.

Only the “psychotropic medication use” variable showed a significant contribution to the model, considering all four domains. The results showed that psychotropic medication use was negatively associated with quality of life in all domains (physical, environment, social relationships, and psychological). For instance, taking psychotropic medications significantly reduces physical quality of life (beta=-0.62, p=0.00001). Thus, psychotropic medication use can negatively impact the perception of quality of life of people who take these medications (Table 4).

**Table 4:** Linear regression model for factors associated with quality of life in the four World Health Organization Quality of Life - brief version domains in patients seen at the mastology outpatient clinic

Variable	Beta	95%CI	*p* value
**Physical**			
Constant	4.05	3.93 a 4.17	0.000
Psychotropic medications	-0.62	-0.89 a -0.35	0.00001
**Environment**			
Constant	4.01	3.90 a 4.11	0.000
Psychotropic medications	-0.28	-0.51 a -0.05	0.016
**Social relationship**			
Constant	4.05	3.95 - 4.16	0.000
Psychotropic medications	-0.28	0.51 a -0.04	0.022
**Psychological**			
Constant	4.13	4.03 a 4.23	0.000
Psychotropic medications	-0.51	-0.73 a -0.29	0.00001

*95%CI - 95% Confidence Interval.*

It was found that 23.6% of the women investigated were using psychotropic medications. Benzodiazepine anxiolytics were most frequently used (45.8%), followed by antidepressants (41.6%). The main indications for medication use were anxiety (38.1%), insomnia (28.6%), and depression (23.8%). It was found that 45% of the women using psychotropic medications had been using them for one to three years. Furthermore, 5.3% were using psychotropic medications without a prescription; 60% received a prescription from a physician; and 94.1% were not undergoing non-pharmacological treatment.

A bivariate analysis revealed a significant association between psychotropic medication use and the “age” (p=0.001), “marital status” (p=0.028), “religion” (p=0.035), “stage of breast cancer” (p=0.008), and “comorbidities” (p=0.019) variables. A logistic regression model was developed to assess the impact of independent variables on the probability of patients in the sample using psychotropic medications. The model was composed of variables that were significant (p<0.05) in the chi-square test (age, marital status, religion, stage, and comorbidities). It was found that only the “religion” and “stage of breast cancer” variables contributed significantly to the model (Table 5).

**Table 5:** Logistic regression model for analyzing factors associated with psychotropic medication use in women with breast cancer

	Variable	Category	OR	95%CI	*p* value
**Psychotropic medication use**	Religion	Catholic	Ref	Ref	Ref
		Non-Catholic	3.27	1.08 - 9.97	0.037
	Stage	I	Ref	Ref	Ref
		II	3.37	0.93 - 12.23	0.064
		III	8.06	1.82 - 35.67	0.006

*OR - Odds Ratio; 95%CI - 95% Confidence Interval.*

The analysis revealed that non-Catholic participants were 3.27 times more likely to use psychotropic medications (OR=3.27; CI=1.08 - 9.97; p=0.037). Women with stage II of the disease were 3.37 times more likely to use psychotropic medications compared to those with stage I (OR=3.37; CI=0.93 - 12.23; p=0.064). Women with stage III were 8.06 times more likely to use psychotropic medications compared to those with stage I (OR=8.06; CI=1.82 - 35.67; p=0.006) (Table 5).

## DISCUSSION

The results of this study revealed that 15.7% of women with breast cancer presented a suicidal risk: a significant prevalence given the oncological context. This finding is consistent with previous studies with breast cancer patients, reinforcing evidence from international literature that reveals a higher risk of suicide in women with breast cancer compared to the general population^(22)^. Additionally, a systematic review of meta-analyses estimated that suicide mortality is 1.5 to 1.7 times higher in cancer patients than in the general population, reinforcing the need for early detection strategies and effective psychosocial support^(23)^.

A study conducted in Japan showed that 51.4% of breast cancer patients diagnosed with major depression presented suicidal ideation, revealing the critical intersection between psychological distress and suicide risk in this population^(24)^. Similarly, another study identified, in a large sample of female breast cancer survivors, a higher likelihood of suicide when compared to the general population^(25)^. These data suggest that, although the five-year survival rate for breast cancer is rising^(3,4)^, the emotional distress and psychosocial impact of the diagnosis remain significant.

A review study indicated that although the literature identifies multiple variables that impact the prevalence of mental disorders after a breast cancer diagnosis, there appears to be a lack of guidance for screening for mental health comorbidities in patients with this diagnosis^(26)^. The aspects described reinforce the need for continuous monitoring of this population’s mental health, and the implementation of screening protocols and psychological support integrated into cancer care to mitigate the risk of suicide in women with breast cancer.

This study identified that women using psychotropic medications had a 7.92 times greater risk of suicide. Previous studies also suggest that the diagnosis and treatment of breast cancer can intensify mental disorders, increasing vulnerability to psychotropic medication use and the risk of suicide^(27,28)^. Hence, research^(29)^ observed a significant increase in the prevalence of anxiety and depression disorders, as well as an increase in psychotropic medication use after a cancer diagnosis, which reinforces the role of this type of medication as a marker of psychological distress.

Early detection of patients vulnerable to suicide risk, especially those using psychotropic medications, is fundamental to enabling more effective interventions in cancer care. Collaboration between oncology and mental health teams can facilitate closer monitoring, contributing to the appropriate management of the effects of these medications on psychological well-being. Furthermore, further research is needed to deepen the understanding of the mechanisms involved and to guide the safe use of these medications in oncological settings.

In this study, mean quality of life scores were lower in the physical and psychological domains. Furthermore, psychotropic medication use was associated with worse quality of life scores in all WHOQOL-bref domains. The literature shows that breast cancer patients frequently present with reduced scores, especially in the physical and psychological domains^(30,31)^. A study of over 2,000 women confirmed poorer physical health indicators among breast cancer survivors, often impacted by treatment-related side effects^(31)^. The adverse effects of psychotropic medications, such as sedation, cognitive impairment, and weight gain, can contribute to a decline in quality of life, in addition to social stigma associated with their use^(32,33)^.

Another relevant finding was the higher prevalence of psychotropic medication use among women in stage III of the disease, reinforcing the association between clinical severity and psychological distress. The study mentions that mental disorders are more prevalent in advanced stages of cancer, but are frequently underdiagnosed, leading to significant impairments in quality of life^(16)^. These aspects highlight the importance of considering specific mental health interventions for women with more advanced stages of breast cancer, as well as a careful assessment of the risks and benefits of using psychotropic medications in the treatment of these women.

The association between religion and psychotropic medication use also emerged as significant in this study, as non-Catholic women were more likely to use these medications. Although the literature on the specific relationship between Catholic religion and psychotropic medication use in women with breast cancer is still scarce, studies indicate that religiosity can positively influence coping with the disease and act as a protective factor for mental health, reducing the perceived need for psychotropic medications^(34,35)^.

Another relevant finding concerns psychotropic medication use duration, as 45% of users had been using them for more than a year, with most prescriptions issued by general practitioners. Furthermore, most participants were not receiving psychological treatment. This pattern suggests a predominance of isolated pharmacological interventions, contradicting recommendations for an integrated approach that includes non-pharmacological therapies, such as psychotherapy^(16,36)^.

In response to this gap, several interventions have been assessed. A recent meta-analysis identified that “empathy therapy” was effective in reducing suicidal ideation and behavior in cancer patients^(37)^. Approaches such as cognitive-behavioral therapy, mindfulness-based interventions, and brief psychotherapy have also shown a positive impact on depressive symptoms, anxiety, fatigue, and spiritual well-being^(36,38,39)^.

The findings of this study reinforce the complexity of cancer care, indicating that suicidal risk and decreased quality of life should not be treated in isolation. An integrative approach is needed, considering clinical, emotional, spiritual, and social aspects, with systematic mental health screening, judicious psychotropic medication use, and the promotion of comprehensive therapeutic interventions, contributing to humanized care and quality of life for women with breast cancer.

### Study limitations

This study has limitations that should be considered when interpreting the results. The cross-sectional design makes it impossible to establish causal relationships among the variables analyzed. Furthermore, the use of non-probabilistic sampling and data collection at a single referral service may introduce selection biases and limit the generalizability of the findings to other populations. Information bias is also possible, since the data were self-reported and involved sensitive topics such as psychological distress and suicidal risk, which are subject to underestimation or overestimation.

Future research should consider longitudinal designs and larger sample sizes, including different geographic and healthcare contexts, to deepen the understanding of the relationships between mental health, psychotropic medication use, and suicide risk in women with breast cancer. Qualitative studies can also contribute to exploring, in greater depth, the subjective experiences related to coping with the disease, supporting more sensitive and integrated interventions in clinical practice.

### Contributions to nursing

The findings of this study highlight the importance of integrating mental health screening as a routine part of nursing care for women with breast cancer. The identification of symptoms of psychological distress and psychotropic medication use in this group reinforce the need for systematic emotional assessment actions, as well as supportive and individualized interventions. For oncology nursing practice, this implies recognizing the complexity of these women’s experiences and acting sensitively, articulating clinical, emotional, and spiritual aspects in the care plan, with a view to promoting quality of life and preventing risks such as suicide.

## CONCLUSIONS

This study showed that the risk of suicide in women with breast cancer was significantly associated with psychotropic medication use, being almost eight times higher among those who used these medications. Furthermore, psychotropic medication use was associated with worse quality of life scores in the physical, psychological, social relationships, and environmental domains. Higher consumption of these medications was also observed among women with stage III cancer and among those who did not identify with the Catholic faith. These results highlight the importance of a broader approach to oncological care that considers not only the clinical condition but also the psychosocial, spiritual, and emotional determinants. For nursing practice, the findings point to the need to incorporate systematic mental health screening strategies, as well as integrated interventions that promote acceptance, qualified listening, and the strengthening of support networks, contributing to the prevention of adverse outcomes and the promotion of these women’s quality of life.

It is recommended to implement routine suicide risk screening at all follow-up appointments, with a defined flow for immediate referral to mental health services, safety plan recording, and active monitoring by nursing staff. It is also recommended to conduct a systematic review of psychotropic medication use and offer psychotherapeutic interventions, in an integrated management approach involving nursing, oncology, mental health, and clinical pharmacy, with periodic reassessment of quality of life outcomes. These measures are applicable in routine care and can contribute to improving quality of life and reducing adverse outcomes.

## Data Availability

The research data are available only upon request.
