# Supplementary material for: Suicide, quality of life, and psychotropic medication use in women with breast cancer
Source: Rev Bras Enferm. 2026 Aug 3;79:e20240573. doi: 10.1590/0034-7167-2024-0573 (PMC13435863; doi:10.1590/0034-7167-2024-0573)
Supplement: Translation

# Translation: Suicídio, qualidade de vida e uso de psicotrópicos em mulheres com câncer de mama

**DOI:** 10.1590/0034-7167-2024-0573pt

**Authors:** Leonardo Felipe Ferreira de Carvalho, Adriana Inocenti Miasso

**Affiliations:** I Universidade de São Paulo. Ribeirão Preto, São Paulo, Brasil

**Keywords:** Estudos Transversais, Suicídio, Psicotrópicos, Qualidade de Vida, Oncologia.

## Abstract

**Objetivos:**

identificar fatores associados ao risco suicida, qualidade de vida e consumo de psicotrópicos em mulheres com câncer de mama atendidas em hospital oncológico.

**Métodos:**

estudo transversal, descritivo correlacional, com abordagem quantitativa, realizado no ambulatório de mastologia de hospital especializado em oncologia. Participaram 102 mulheres que responderam a questionários específicos. Utilizaram-se modelos de regressão linear e logística para avaliar a associação entre o uso de psicotrópicos, risco suicida e qualidade de vida.

**Resultados:**

15,7% das participantes apresentaram risco suicida, e 23,6% usavam psicotrópicos, principalmente benzodiazepínicos e antidepressivos. Mulheres não católicas e em estágio III da doença apresentaram maior consumo de psicotrópicos. A qualidade de vida foi menor nos domínios físico e psicológico, e o uso de psicotrópicos se associou a piores escores em todos os domínios.

**Conclusões:**

os achados ressaltam a importância da identificação precoce do risco suicida e estratégias personalizadas para apoio emocional para mulheres com câncer de mama.

## INTRODUÇÃO

O suicídio é uma das principais causas de morte globalmente, representando um problema significativo de saúde pública. De acordo com estimativas recentes da Organização Mundial da Saúde (OMS), mais de 700.000 pessoas morrem por suicídio a cada ano, o que equivale a uma morte a cada 40 segundos, destacando a necessidade urgente de estratégias de prevenção eficazes, especialmente em populações vulneráveis, como as mulheres diagnosticadas com câncer de mama^(1)^.

O câncer de mama é o tipo de câncer mais comum entre as mulheres, com uma incidência crescente impulsionada pelo envelhecimento populacional e mudanças nos estilos de vida^(2)^.

O Instituto Nacional de Câncer projeta que o Brasil registre 73.610 novos casos da doença em 2024, com uma taxa de 66,54 casos por 100 mil mulheres^(3,4)^. Pesquisas mostram que um diagnóstico de câncer de mama não causa apenas sofrimento físico significativo, mas também sofrimento psicológico grave, especialmente nos primeiros anos após o diagnóstico, quando os pacientes enfrentam intensos conflitos emocionais e psicológicos, o que tem sido sugerido aumentar o risco de suicídio^(5)^.

A carga psicológica enfrentada por mulheres com câncer de mama é multifacetada. A ansiedade sobre a mortalidade iminente, as alterações na imagem corporal devido a cirurgias e tratamentos, e o estigma associado à doença são fatores que contribuem significativamente para o aumento do risco suicida^(6-11)^. Além disso, a depressão e ansiedade são prevalentes nessa população, com taxas significativamente mais altas do que na população geral_._ A depressão, em particular, é um fator de risco conhecido para o suicídio, podendo afetar negativamente a adesão ao tratamento e a qualidade de vida^(7,11)^.

O manejo do câncer de mama frequentemente envolve o uso de psicotrópicos para tratar sintomas psicológicos secundários, como depressão e ansiedade^(12,13)^. Esses medicamentos, embora eficazes na melhoria da qualidade de vida, apresentam desafios relacionados a interações medicamentosas e efeitos colaterais potenciais. Além disso, o estigma associado ao uso de psicotrópicos pode influenciar negativamente a disposição das pacientes em buscar tratamento^(14)^.

O aumento projetado nos casos de câncer de mama pode ultrapassar 2 milhões anualmente no mundo todo até 2025. Com isso, destaca-se a necessidade de abordagens de saúde integrativas que não apenas tratem a doença física, mas também ofereçam suporte emocional e psicológico abrangente^(15,16)^.

Estratégias de intervenção que combinam tratamento farmacológico com terapias psicossociais têm mostrado resultados promissores na redução dos sintomas de depressão e ansiedade, melhorando a qualidade de vida das pacientes^(17,18)^.

## OBJETIVOS

Identificar fatores associados ao risco suicida, qualidade de vida e consumo de psicotrópicos em mulheres com câncer de mama em hospital oncológico.

## MÉTODOS

### Aspectos éticos

O estudo respeitou os preceitos éticos da pesquisa com seres humanos, sendo aprovado pelo Comitê de Ética em Pesquisa da instituição proponente. Os sujeitos foram informados sobre o anonimato e a liberdade em aceitar ou recusar a participação na pesquisa. Todas as participantes que aceitaram participar do estudo assinaram o Termo de Consentimento Livre e Esclarecido.

### Referencial teórico

As relações entre variáveis específicas foram analisadas segundo os referenciais descritos nas diretrizes de pesquisa em saúde mental e qualidade de vida da OMS, e instrumentos padronizados, como o *Mini International Neuropsychiatric Interview* (MINI) e o *World Health Organization Quality of Life - brief version* (WHOQOL-bref).

### Tipo de estudo

Trata-se de estudo observacional, do tipo transversal, com abordagem quantitativa e caráter exploratório, realizado de acordo com as diretrizes da declaração *STrengthening the Reporting of OBservational studies in Epidemiology*. O desenho transversal permite a análise simultânea de exposição e desfecho em um determinado momento, possibilitando a identificação de associações entre variáveis, embora não permita inferir causalidade.

### Cenário do estudo

O estudo foi realizado no ambulatório de mastologia de hospital oncológico localizado na região Sudeste do Brasil. Esse ambulatório oferece atendimento ao paciente desde seu diagnóstico, seguindo seu tratamento cirúrgico, quimioterápico, radioterápico e reabilitação, e, se necessário, cuidados paliativos e terminalidade. O serviço conta com um grupo interdisciplinar que envolve radiologistas, mastologistas, médicos nucleares, geneticista, oncologistas, radio-oncologistas, patologistas, enfermeiros, fisioterapeutas, nutricionistas, psicólogos e todos os demais profissionais que possam contribuir para o tratamento ideal de cada paciente.

### População e amostra

A população do estudo foi constituída por mulheres que realizavam acompanhamento no ambulatório de mastologia de hospital oncológico público da região Sudeste do Brasil. Para determinar amostra, foi realizado cálculo amostral por estatístico do Núcleo de Epidemiologia e Bioestatística do referido hospital. O cálculo amostral foi realizado utilizando como referência os dados de estudo^(19)^ que aplicou o WHOQOL-bref em mulheres com câncer de mama. Consideraram-se um nível de confiança de 95% e um erro absoluto tolerável de 1 ponto nos escores do referido instrumento, estimando-se, assim, a necessidade de uma amostra mínima de 102 participantes, quantitativo que foi atingido no período de coleta. Esse cálculo seguiu a fórmula n=(Z·σ/d)^2, com Z=1,96, erro absoluto d=1 ponto e o desvio padrão do domínio mais variável do WHOQOL-bref no estudo de referência, o que justifica a amostra mínima de 102. Esse procedimento assegurou maior precisão na estimativa da média. Ainda assim, reconhece-se que se trata de estudo exploratório, sujeito às limitações inerentes ao número de participantes.

Foram critérios de inclusão ser mulher diagnosticada com qualquer tipo de câncer de mama, ter idade igual ou superior a 18 anos, ser capaz de se comunicar verbalmente me português e estar em acompanhamento no ambulatório de mastologia do hospital em estudo. Foi considerado critério de exclusão não responder às perguntas dos instrumentos de pesquisa em sua completude.

### Procedimentos metodológicos

Para coleta de dados, foram realizadas visitas ao ambulatório de mastologia no período de março a julho de 2024. As pacientes que compareceram ao ambulatório para atendimento e que preenchiam os critérios de inclusão foram convidadas a participar da pesquisa. As participantes que aceitaram participar receberam informações detalhadas sobre o estudo, e após assinarem o Termo de Consentimento Livre e Esclarecido, participaram de entrevista conduzida por pesquisador do estudo. As entrevistas foram realizadas em consultórios do ambulatório de mastologia, com duração média de 30 minutos. Para realização das entrevistas, foram utilizados um questionário estruturado, elaborado pelos pesquisadores e com validação de conteúdo por juízes, e instrumentos já validados.

O questionário utilizado abordou variáveis sociodemográficas, clínicas e relacionadas ao uso de psicotrópicos, incluindo idade, escolaridade, estado civil, religião, situação de trabalho/ocupação, renda mensal individual e/ou familiar, número de moradores no domicílio, local de acometimento primário do câncer, tempo de tratamento oncológico, histórico de cirurgias oncológicas, presença de outras enfermidades, e uso contínuo de medicamentos não psicotrópicos. Para coleta de dados referentes à variável desfecho “uso de psicotrópicos”, foi incluída neste instrumento a questão “utiliza psicotrópicos?”, com possibilidade de respostas “sim” e “não”.

Para avaliação do risco de suicídio, foi utilizado o MINI, versão 5.0.0, Módulo C - Risco de Suicídio. O MINI é um instrumento estruturado e validado internacionalmente, adaptado à população brasileira^(20)^. Apresenta alta sensibilidade e especificidade, além de boa confiabilidade, o que o torna adequado para uso em contextos clínicos^(20)^, incluindo pacientes oncológicos, por sua capacidade de identificar sintomas emocionais relevantes de forma rápida e padronizada. O questionário é organizado por módulos diagnósticos independentes, elaborados de forma a otimizar a sensibilidade do instrumento. As respostas consistem em variável dicotômica: sim ou não. Os módulos são identificados por letras, e cada letra corresponde a uma categoria diagnóstica^(20)^. Considerando os objetivos da presente pesquisa, foi utilizado o Módulo C - Risco de Suicídio.

A qualidade de vida foi avaliada por meio do WHOQOL-bref, que engloba quatro domínios: físico; psicológico; relações sociais; e meio ambiente. O WHOQOL-bref foi desenvolvido pela OMS em parceria com 20 centros de estudos localizados em 18 países, e foi validado no Brasil^(21)^, apresentando alta consistência e boa validade de construto e discriminante. Os resultados obtidos no instrumento são classificados conforme a média das respostas, de acordo com a seguinte escala: necessita melhorar (quando for 1 até 2,9); regular (3 até 3,9); boa (4 até 4,9); e muito boa (5). Revisão integrativa que investigou a confiabilidade do WHOQOL-BREF em pesquisas com mulheres diagnosticadas com câncer de mama apontou que o questionário apresenta níveis adequados de consistência interna. Os resultados indicaram que os valores do alfa de Cronbach para os diferentes domínios da qualidade de vida permanecem estáveis, independentemente do estágio da doença, reforçando a utilidade do instrumento nessa população específica^(21)^.

A confidencialidade dos participantes foi assegurada por meio da realização das entrevistas em local reservado, da codificação dos dados sem identificação nominal e do armazenamento das informações em ambiente eletrônico protegido, com acesso restrito à equipe de pesquisa.

A coleta de dados foi interrompida quando se atingiu o tamanho amostral previsto de 102 participantes. As pacientes detectadas com risco de suicídio foram encaminhadas para o Departamento de Psico-Oncologia do hospital em estudo para avaliação médica e acompanhamento psicológico e psiquiátrico.

### Análise dos dados

Para análise dos dados, foi utilizada a abordagem quantitativa. Os dados foram coletados com auxílio de um computador durante a entrevista e armazenados utilizando a plataforma REDCAp. Posteriormente, os dados foram transportados para o programa *Statistical Package for the Social Sciences*, versão 21.5, para análise. Os pesquisadores se comprometeram a coletar e armazenar os dados, em consonância com a Lei Geral de Proteção de Dados Pessoais. Para abordagem do risco suicida e uso de psicotrópicos como variáveis dependentes, foram realizadas as análises univariada (teste qui-quadrado e, em casos de categorias com menos de cinco indivíduos, foi utilizado o teste exato de Fisher) e regressão logística multivariada. Para análise da qualidade de vida, foram utilizados teste t de *Student* e regressão linear múltipla. Foram consideradas significativas as associações nas quais p≤0,05. Além das comparações entre grupos, exploramos a correlação entre risco suicida e os domínios do WHOQOL-bref por meio de correlação ponto-bisserial; quando indicado, utilizamos correlação de Spearman.

## RESULTADOS

Participaram do estudo 102 mulheres com câncer de mama. A maioria estava na faixa etária de 41 a 59 anos (53,9%), possuía ensino médio (51%), tinha companheiro (67,6%), estava sem ocupação (62,7%), tinha renda menor ou igual a três salários mínimos (62,7%) e não morava sozinha (88,2%). Houve maior porcentagem de participantes da região Nordeste (35,3%) e não católicas (50%). Em relação às características clínicas, verificou-se que a maior porcentagem de participantes estava no estadiamento I da doença (44,6%), com comorbidades (50,9%) e em uso de medicamentos não psicotrópicos (55,9%) (Tabela 1).

**Tabela 1:** Características sociodemográficas e clínicas das mulheres com câncer de mama atendidas em ambulatório de mastologia de hospital oncológico brasileiro (N = 102)

Variáveis	n	%
Idade (anos)		
18-40	3	2,9
41-59	55	53,9
Igual ou superior a 60	43	42,2
*Missing*	1	0,9
Total	102	100
Região		
Norte	22	21,6
Nordeste	36	35,3
Centro-Oeste	4	3,9
Sudeste	32	31,4
Sul	5	4,9
*Missing*	3	2,9
Total	102	100,0
**Escolaridade**		
Analfabeto	2	2,0
Fundamental	21	20,6
Médio	52	51,0
Superior	27	26,5
Total	102	100
**Estado civil**		
Sem companheiro	33	32,4
Com companheiro *Missing*	69	67,6
Total	102	100
**Religião**		
Católica	49	48,0
Não católica	51	50,0
*Missing*	2	2,0
Total	102	100,0
**Ocupação**		
Sem ocupação	64	62,7
Com ocupação	36	35,3
*Missing*	2	2,0
Total	102	100,0
**Renda**		
≤ 3 salários mínimos	64	62,7
> 3 salários mínimos	38	37,3
Total	102	100,0
**Mora sozinho**		
Não	90	88,2
Sim	9	8,8
*Missing*	3	3,0
Total	102	100,0
**Estágio**		
I	45	44,6
II	40	39,6
III	17	15,8
Total	102	100,0
**Comorbidades**		
Não	45	44,1
Sim	52	50,9
*Missing*	5	5,0
Total	102	100,0
**Uso de medicamentos não psicotrópicos**		
Não	45	44,1
Sim	57	55,9
Total	102	100,0

Constatou-se, neste estudo, por meio do MINI, que 15,7% das participantes foram classificadas com risco para suicídio e 43,8% não possuíam risco para suicídio. Para realização do teste qui-quadrado, a variável “risco suicida” foi classificada em “com risco” e “sem risco”. A análise univariada mostrou que nenhuma das variáveis sociodemográficas investigadas foi associada ao risco suicida. Em relação às características clínicas e terapêuticas, houve associação significativa entre uso de psicotrópicos e risco suicida (p=0,001). Constatou-se que, entre as mulheres com risco suicida, a maioria estava em uso de psicotrópicos (56,2%).

A Tabela 2 apresenta um modelo de regressão logística que investiga a relação entre o risco suicida em pacientes com câncer de mama e o uso de psicotrópicos. Os resultados mostram uma correlação significativa entre o uso de psicotrópicos e um risco maior de suicídio. Houve um *Odds Ratio* (OR) de 7,92 e um Intervalo de Confiança (IC) de 95% entre 2,5 e 25,3 (p=0,0005). Isso indica que pacientes que utilizam medicamentos psicotrópicos têm uma probabilidade maior de suicídio do que aqueles que não utilizam.

**Tabela 2:** Modelo de regressão logística para análise de fatores associados do risco suicida em pacientes com câncer de mama

	Variável	Categoria	OR	IC95%	Valor de *p*
**Risco suicida**	Uso de medicamentos psicotrópicos	Não	Ref	Ref	Ref
		Sim	7,92	2,5 - 25,3	0,0005

*OR - Odds Ratio; IC95% - Intervalo de Confiança de 95%.*

Constatou-se que os escores médios de qualidade de vida foram menores nos domínios físico (3,7) e psicológico (3,8), e maiores nos domínios relações sociais (4,0) e meio ambiente (4,0). Nos domínios físico e psicológico, a maioria das mulheres foi classificada com qualidade de vida regular (72,5% e 69,6%, respectivamente). Nos domínios relações sociais e meio ambiente, a maioria foi classificada com qualidade de vida boa (72,5% em ambos os domínios) (Tabela 3).

**Tabela 3:** Análise dos desempenhos categorizados de acordo com os domínios do *World Health Organization Quality of Life - brief version* e por média

Variáveis	Necessita melhorar		Regular		Boa		Muito Boa		Média
	n	%	n	%	n	%	n	%	
Qualidade de vida - desempenhos									
Físico	7	6,9	74	72,5	21	20,6	0	0	3,7
Psicológico	6	5,9	71	69,6	22	21,6	3	2,9	3,8
Relações sociais	2	2,0	15	14,7	74	72,5	11	10,8	4,0
Meio ambiente	3	2,9	22	21,6	74	72,5	3	2,9	4,0

Na análise da associação entre risco suicida e os domínios de qualidade de vida, não foram observadas diferenças estatisticamente significativas. Ainda assim, foi possível identificar alguns padrões descritivos, sugerindo que pacientes com maior risco suicida tendiam a relatar piores escores em determinados domínios. Foi realizado modelo de regressão linear para identificar o impacto das variáveis independentes nos diferentes domínios do WHOQOL-bref. No modelo, foram incluídas todas as variáveis que apresentaram associação significativa (p<0,05) na análise univariada. No domínio físico, houve associação com as variáveis “estágio do câncer de mama (p=0,008)” e “uso de medicamentos psicotrópicos” (p=0,001). No domínio psicológico, obteve-se associação com escolaridade (p=0,034), estágio do câncer de mama (p=0,002), comorbidades (p=0,020) e uso de medicamentos psicotrópicos (p<0,001). No domínio relações sociais, mostraram-se associadas as variáveis “estágio do câncer de mama” (p=0,031) e “uso de medicamentos psicotrópicos” (p=0,022). Houve associação significativa entre a classificação da qualidade de vida no domínio meio ambiente e a variável “uso de medicamentos psicotrópicos” (p=0,006).

Apenas a variável “uso de medicamento psicotrópico” apresentou contribuição significativa para o modelo, considerando os quatro domínios. Os resultados mostraram que o uso de medicamentos psicotrópicos esteve negativamente associado à qualidade de vida em todos os domínios (físico, meio ambiente, relações sociais e psicológico). Por exemplo, tomar medicamentos psicotrópicos reduz significativamente a qualidade de vida física (beta=-0,62, p=0,00001). Desse modo, o uso de medicamentos psicotrópicos pode impactar negativamente a percepção da qualidade de vida das pessoas que tomam esses medicamentos (Tabela 4).

**Tabela 4:** Modelo de regressão linear para fatores associados à qualidade de vida nos quatro domínios do *World Health Organization Quality of Life - brief version* em pacientes atendidas no ambulatório de mastologia

Variável	Beta	IC95%	Valor de *p*
**Físico**			
Constante	4,05	3,93 a 4,17	0,000
Psicotrópicos	-0,62	-0,89 a -0,35	0,00001
**Meio ambiente**			
Constante	4,01	3,90 a 4,11	0,000
Psicotrópicos	-0,28	-0,51 a -0,05	0,016
**Relação social**			
Constante	4,05	3,95 - 4,16	0,000
Psicotrópicos	-0,28	0,51 a -0,04	0,022
**Psicológico**			
Constante	4,13	4,03 a 4,23	0,000
Psicotrópicos	-0,51	-0,73 a -0,29	0,00001

*IC95% - Intervalo de Confiança de 95%.*

Constatou-se que 23,6% das mulheres investigadas estavam em uso de medicamentos psicotrópicos. Houve maior frequência de uso de ansiolíticos benzodiazepínicos (45,8%), seguidos dos antidepressivos (41,6%). As indicações principais para o uso de fármacos foram ansiedade (38,1%), insônia (28,6%) e depressão (23,8%). Verificou-se que 45% das mulheres em uso de psicotrópicos apresentavam um tempo de uso de um a três anos. Além disso, 5,3% faziam uso de medicamentos psicotrópicos sem prescrição médica; 60% receberam a prescrição de um clínico; e 94,1% não realizavam tratamento não farmacológico.

A análise bivariada revelou associação significativa entre o uso de medicamentos psicotrópicos e as variáveis “idade” (p=0,001), “estado civil” (p=0,028), “religião” (p=0,035), “estágio do câncer de mama” (p=0,008) e “comorbidades” (p=0,019). Foi elaborado modelo de regressão logística para avaliar o impacto das variáveis independentes na probabilidade de as pacientes da amostra utilizarem medicamentos psicotrópicos. O modelo foi composto por variáveis que se apresentaram significativas (p<0,05) no teste qui-quadrado (idade, estado civil, religião, estágio e comorbidades). Constatou-se que apenas as variáveis “religião” e “estágio do câncer de mama” contribuíram significativamente para o modelo (Tabela 5).

**Tabela 5:** Modelo de regressão logística para análise de fatores associados ao uso de psicotrópicos em mulheres com câncer de mama

	Variável	Categoria	OR	IC95%	Valor de *p*
**Uso de psicotrópicos**	Religião	Católico	Ref	Ref	Ref
		Não católico	3,27	1,08 - 9,97	0,037
	Estágio	I	Ref	Ref	Ref
		II	3,37	0,93 - 12,23	0,064
		III	8,06	1,82 - 35,67	0,006

*OR - Odds Ratio; IC95% - Intervalo de Confiança de 95%.*

A análise revelou que as participantes não católicas tiveram 3,27 vezes mais chances de uso de medicamentos psicotrópicos (OR=3,27; CI=1,08 - 9,97; p=0,037). Mulheres com estágio II da doença tinham 3,37 vezes mais chances de uso de psicotrópicos em comparação àquelas com estágio I (OR=3,37; CI=0,93 - 12,23; p=0,064). Mulheres com estágio III tinham 8,06 vezes mais chances de usar medicamentos psicotrópicos em comparação às com estágio I (OR=8,06; CI=1,82 - 35,67; p=0.006) (Tabela 5).

## DISCUSSÃO

Os resultados deste estudo revelaram que 15,7% das mulheres com câncer de mama apresentaram risco suicida: uma prevalência relevante diante do contexto oncológico. Este achado está em consonância com estudos prévios com pacientes com câncer de mama, reforçando evidências da literatura internacional que revelam risco de suicídio maior em mulheres com câncer de mama em comparação à população geral^(22)^. Complementarmente, em revisão sistemática de meta-análises, estimou-se que a mortalidade por suicídio é entre 1,5 e 1,7 vezes mais elevada em pacientes com câncer do que na população em geral, o que reforça a necessidade de estratégias de detecção precoce e apoio psicossocial efetivo^(23)^.

Estudo realizado no Japão mostrou que 51,4% das pacientes com câncer de mama e diagnóstico de depressão maior apresentavam ideação suicida, revelando a interseção crítica entre sofrimento psíquico e o risco suicida nesta população^(24)^. De forma semelhante, outra pesquisa identificou, em ampla amostra de mulheres sobreviventes de câncer de mama, maior probabilidade de suicídio quando comparadas à população geral^(25)^. Esses dados sugerem que, embora a taxa de sobrevida em cinco anos para o câncer de mama esteja em ascensão^(3,4)^, o sofrimento emocional e o impacto psicossocial do diagnóstico permanecem expressivos.

Estudo de revisão apontou que apesar de a literatura identificar múltiplas variáveis que impactam a prevalência de transtornos mentais após o diagnóstico de câncer de mama, parece haver falta de orientação para o rastreio de comorbilidades de saúde mental em pacientes com o diagnóstico^(26)^. Os aspectos descritos reforçam a necessidade de vigilância contínua da saúde mental dessa população, e implementação de protocolos de rastreamento e suporte psicológico integrados ao cuidado oncológico para mitigar o risco de suicídio em mulheres com câncer de mama.

O presente estudo identificou que mulheres em uso de psicotrópicos apresentaram chance 7,92 vezes maior de risco suicida. Estudos anteriores também sugerem que o diagnóstico e tratamento do câncer de mama podem intensificar transtornos mentais, aumentando a vulnerabilidade para o uso de psicotrópicos e o risco de suicídio^(27,28)^. Nesta direção, pesquisa^(29)^ observou aumento significativo na prevalência de transtornos de ansiedade e depressão, bem como aumento no uso de psicotrópicos após o diagnóstico de câncer, o que reforça o papel desse tipo de medicação como marcador de sofrimento psíquico.

A detecção precoce de pacientes vulneráveis ao risco suicida, em especial aquelas que fazem uso de psicotrópicos, é fundamental para viabilizar intervenções mais eficazes no cuidado oncológico. A articulação entre equipes de oncologia e saúde mental pode favorecer um acompanhamento mais próximo, contribuindo para o manejo adequado dos efeitos desses medicamentos sobre o bem-estar psíquico. Ademais, novas pesquisas são necessárias para aprofundar a compreensão dos mecanismos envolvidos e orientar o uso seguro desses medicamentos em contextos oncológicos.

Neste estudo, os escores médios de qualidade de vida foram mais baixos nos domínios físico e psicológico. Além disso, o uso de psicotrópicos se associou a piores escores de qualidade de vida em todos os domínios do WHOQOL-bref. A literatura mostra que pacientes com câncer de mama frequentemente apresentam escores reduzidos especialmente nos domínios físico e psicológico^(30,31)^. Em estudo com mais de 2.000 mulheres, confirmaram-se piores indicadores de saúde física entre sobreviventes de câncer de mama, frequentemente impactadas por sequelas do tratamento^(31)^. Os efeitos adversos dos psicotrópicos, como sedação, comprometimento cognitivo e ganho de peso, podem contribuir para o declínio na qualidade de vida, além do estigma social associado ao seu uso^(32,33)^.

Outro achado relevante foi a maior prevalência de uso de psicotrópicos entre mulheres em estágio III da doença, o que reforça a associação entre a gravidade clínica e o sofrimento psíquico. Estudo menciona que transtornos mentais são mais prevalentes em estágios avançados do câncer, porém frequentemente subdiagnosticados, o que acarreta prejuízos significativos à qualidade de vida^(16)^. Tais aspectos apontam para a importância de considerar intervenções específicas de saúde mental para mulheres com câncer de mama em estágios mais avançados, bem como a avaliação criteriosa do risco e benefício do uso de psicotrópicos no tratamento dessas mulheres.

A associação entre religião e uso de psicotrópicos também emergiu como significativa neste estudo, pois as mulheres não católicas apresentaram maior chance de fazer uso desses medicamentos. Embora a literatura sobre a relação específica entre religião católica e uso de psicotrópicos em mulheres com câncer de mama ainda seja escassa, estudos apontam que a religiosidade pode influenciar positivamente o enfrentamento da doença e atuar como fator de proteção à saúde mental, reduzindo a necessidade percebida de medicamentos psicotrópicos^(34,35)^.

Outro dado relevante diz respeito à duração do uso de medicamentos psicotrópicos, pois 45% das usuárias faziam uso há mais de um ano, sendo a maioria das prescrições realizadas por médicos clínicos. Além disso, a maioria das participantes não estava em acompanhamento psicológico. Esse padrão sugere predomínio de intervenções farmacológicas isoladas, contrariando recomendações para abordagem integrada que envolva terapias não farmacológicas, como a psicoterapia^(16,36)^.

Em resposta a essa lacuna, várias intervenções vêm sendo avaliadas. Em meta-análise recente, identificou-se que a “*empathy therapy*” foi eficaz na redução da ideação e comportamento suicida em pacientes com câncer^(37)^. Abordagens como a terapia cognitivo-comportamental, intervenções baseadas em *mindfulness* e psicoterapia breve também têm mostrado impacto positivo em sintomas depressivos, ansiedade, fadiga e bem-estar espiritual^(36,38,39)^.

Os achados deste estudo reforçam a complexidade do cuidado oncológico, indicando que o risco suicida e a piora na qualidade de vida não devem ser tratados de forma isolada. É necessária uma abordagem integrativa, que considere aspectos clínicos, emocionais, espirituais e sociais, com triagem sistemática em saúde mental, uso criterioso de psicotrópicos e promoção de intervenções terapêuticas abrangentes, contribuindo para o cuidado humanizado e a qualidade de vida de mulheres com câncer de mama.

### Limitações do estudo

Este estudo apresenta limitações que devem ser consideradas na interpretação dos resultados. O delineamento transversal impossibilita estabelecer relações de causalidade entre as variáveis analisadas. Além disso, a utilização de amostragem não probabilística e a coleta em um único serviço de referência podem introduzir vieses de seleção e limitar a generalização dos achados para outras populações. Também é possível a ocorrência de viés de informação, uma vez que os dados foram autorreferidos e envolveram temas sensíveis, como sofrimento psíquico e risco suicida, sujeitos à sub ou superestimação.

Futuras investigações devem considerar delineamentos longitudinais e amostragens mais amplas, incluindo diferentes contextos geográficos e assistenciais para aprofundar a compreensão das relações entre saúde mental, uso de medicamentos psicotrópicos e risco de suicídio em mulheres com câncer de mama. Estudos qualitativos também podem contribuir para explorar, com maior profundidade, as experiências subjetivas relacionadas ao enfrentamento da doença, subsidiando intervenções mais sensíveis e integradas na prática clínica.

### Contribuições para a área da enfermagem

Os achados deste estudo evidenciam a importância de integrar a triagem em saúde mental como parte rotineira do cuidado de enfermagem às mulheres com câncer de mama. A identificação de sintomas de sofrimento psíquico e o uso de medicamentos psicotrópicos neste grupo reforçam a necessidade de ações sistemáticas de avaliação emocional, além de intervenções acolhedoras e individualizadas. Para prática da enfermagem oncológica, isso implica reconhecer a complexidade das vivências dessas mulheres e atuar de forma sensível, articulando aspectos clínicos, emocionais e espirituais no plano de cuidado, com vistas à promoção da qualidade de vida e prevenção de riscos, como o suicídio.

## CONCLUSÕES

Este estudo evidenciou que o risco de suicídio em mulheres com câncer de mama esteve significativamente associado ao uso de medicamentos psicotrópicos, sendo quase oito vezes maior entre aquelas que utilizavam esses medicamentos. Além disso, o uso de psicotrópicos se mostrou associado a piores escores de qualidade de vida nos domínios físico, psicológico, relações sociais e meio ambiente. Verificou-se, também, maior consumo desses medicamentos entre mulheres com câncer em estágio III e entre aquelas que não se identificavam com a fé católica. Tais resultados destacam a importância de uma abordagem ampliada no cuidado oncológico que considere não apenas a condição clínica, mas também os determinantes psicossociais, espirituais e emocionais. Para a prática da enfermagem, os achados apontam para a necessidade de incorporar estratégias sistemáticas de triagem em saúde mental, além de intervenções integradas que favoreçam o acolhimento, a escuta qualificada e o fortalecimento de redes de apoio, contribuindo para prevenção de desfechos adversos e promoção da qualidade de vida dessas mulheres.

Recomenda-se implementar triagem rotineira de risco de suicídio em todas as consultas de seguimento, com fluxo definido para encaminhamento imediato à saúde mental, registro do plano de segurança e monitoramento ativo pela enfermagem. Recomendam-se, ainda, a revisão sistemática do uso de psicotrópicos e oferta de intervenções psicoterapêuticas, em manejo integrado entre enfermagem, oncologia, saúde mental e farmácia clínica, com reavaliação periódica dos desfechos de qualidade de vida. Essas medidas são aplicáveis na rotina assistencial, podendo contribuir para melhorar a qualidade de vida e reduzir desfechos adversos.

## Data Availability

Os dados de pesquisa só estão disponíveis mediante solicitação.
